# Assessment of attitude towards e-professionalism: Students’ perspectives from a Private Medical College in Lahore, Pakistan

**DOI:** 10.12669/pjms.41.1.8644

**Published:** 2025-01

**Authors:** Huma Saeed Khan, Muhammad Ali Rabbani, Faiza Ikram

**Affiliations:** 1 Huma Saeed Khan University of Health Sciences Lahore, Lahore, Pakistan. CMH Lahore Medical College and Institute of Dentistry (IOD), Lahore, Pakistan; 2 Muhammad Ali Rabbani CMH Multan Institute of Medical Sciences, Multan, Pakistan; 3 Faiza Ikram CMH Multan Institute of Medical Sciences, Multan, Pakistan

**Keywords:** E-professionalism, Digital healthcare, Online behaviour, Professionalism in digital age

## Abstract

**Objective:**

This study was conducted to investigate the social media practices and attitudes towards e-professionalism among undergraduate medical students in a medical college of Pakistan.

**Methods:**

This cross-sectional study was conducted on 220 undergraduate medical students from 2^nd^ to final-year MBBS, at CMH Lahore Medical College from March to August 2022. After ethical approval, a printed questionnaire was distributed among students, selected by stratified random sampling technique. Data on demographics, social media usage, and attitudes toward e-professionalism was collected using the pre-validated SMePROF scale. Chi-squared, Fisher’s exact test, and the Mann-Whitney U test were used to analyze the results.

**Results:**

Responses from 220 students were analyzed. Most students were active social media users, spending 5.5±4.38 hours daily on Instagram (82.3%) and Facebook (80%). Male students were less likely to accept friend requests from the patients than females (p=0.009). While overall responsible online behavior was observed, attitudes towards e-professionalism varied. Pre-Clinical students were more likely to post pictures from the workplace than Clinical students (p=0.009). Females majorly supported social media bans (p=0.018), while males were more interested in keeping up with trends (p=0.022). Pre-clinical students favored more freedom in using social media for patient interaction (p=0.034).

**Conclusion:**

This study highlights the widespread social media usage among medical students and its impact on e-professionalism. Gender and academic seniority influence social media practices and e-professionalism. These findings emphasize the importance of targeted education and policies to promote more responsible use of social media and e-professionalism in the health sector.

## INTRODUCTION

Professionalism is a fundamental aspect of healthcare facilitating effective doctor-patient interactions.^1^ It has three broad domains: professional behavior and ethical principles (PROF-1), accountability and conscientiousness (PROF-2), and self-awareness and help-seeking (PROF-3).^2^ With the advent of digitalization and the increased use of social media platform in health sector, the concept of professionalism has expanded into the digital realm, giving rise to the term ‘e-professionalism’.

E-professionalism refers to the behaviors displayed by professionals through digital media, aligning with the traditional paradigms of professionalism.^3^ As healthcare becomes increasingly digitalized, it is imperative to address the impact of digitalization on healthcare practices and the integration of technologies into the professional context.^4^

The role of online media in continuity of care and access to medical services has become evident, particularly during and after the COVID-19 pandemic.^5^ Healthcare professionals now rely more heavily on online media for communication and interaction. The digitalization of the healthcare sector takes various forms, such as mobile health (mHealth), health information technology (IT), wearable devices, telehealth, telemedicine, and online health portals, etc.^4^ Telemedicine and virtual consultations have gained prevalence, enabling provision of remote care and advice to patients. While these advancements have potential benefits, they also introduce new challenges related to e-professionalism.^6^ A systematic review of 44 studies in 2021 identified significant challenges to digitalization in healthcare, including a lack of educational and assessment strategies for digital professionalism and the blurring of personal and professional identities.^6^

The use of technology has blurred the line between professional and personal lives of health professionals, making it increasingly challenging to maintain clear boundaries.^7^ The ability to collect and share information online requires health professionals to be mindful of their online presence and conduct.^8^ E-professionalism entails deploying privacy settings, avoiding inappropriate online behavior, and understanding the potential impact of online communication on professional reputation.^7^,^8^

Studies that examined the role of e-professionalism among medical students indicate both the benefits and dangers of social media use in relation to e-professionalism.^6^,^9^ While there is a positive attitude towards social media use among medical professionals, concerns exist regarding unprofessional content, patient privacy, and interactions with patients and faculty members online.^9^ These studies highlight the necessity for robust e-professionalism training for students.

A review of the literature reveals that demographic factors, such as gender, seniority, and residence in rural or urban areas, can influence e-professionalism among medical students.^3^,^4^ Female students tend to use social media more in professional and academic context as compared to males using it for personal objectives.^10^ Also the increasing age of healthcare professionals is inversely related to social media use.^3^ Understanding these factors can help tailor educational interventions and policies to address the specific needs and challenges.

A study conducted in Pakistan found poor professionalism among young doctors, characterized by rigidity of opinions, unacceptability of contrasting perspectives, and inappropriate use of social media during duty hours.^11^ A study conducted in Karachi revealed the percentage of common social media sites used by the students, but it did not employ a validated questionnaire to assess the various facets of e-professionalism.^12^ Researchers from Lahore in 2021, suggested that medical professionals may unknowingly expose themselves to risks related to e-professionalism lapses due to less vigilant use of social media.^8^

Given the significance of e-professionalism in the healthcare profession, it is crucial to conduct further research to understand its impact on undergraduate medical students and develop targeted interventions and policies that promote responsible online behavior. The objective of this study was to investigate the attitudes of undergraduate medical students toward e-professionalism. Additionally, it seeks to determine the amount of time spent on social media platforms daily and to explore potential associations between time spent on social media, gender, and seniority.

## METHODS

This cross-sectional study was undertaken at CMH Lahore Medical College. The study was conducted from March 2022 to August 2022. The undergraduate medical students ranging from the second to the final year of MBBS were enrolled. Participants were assured that their responses would remain confidential and not influence their academic results. Participation was entirely voluntary, and no incentives were provided. Withdrawn consent and improperly completed forms led to removal of the subject from the study.

### Ethical Approval:

The study was approved by the institute’s Ethical Review Committee (IERC# 660/ERC/CMH/LMC).

The Raosoft^®^ Sample Size Calculator determined a sample size of 187, considering a 50% expected response distribution, 5% margin of error, 90% confidence level and the population size of 600. We distributed 300 forms among the students using stratified random sampling. Stratification was done to ensure equal representation from males and females, as well as from preclinical (2^nd^ and 3^rd^ Year) and clinical (4^th^ and Final Year) students. In each stratum, 75 students were selected by a random number generator.

Data was collected via a printed questionnaire having two sections. The first section collected information about participants’ demographics (including age, gender and year of study) and their social media usage habits via six questions on the categorical response scale (Yes/No), (Annexure-1). The second section used a pre-validated scale called the Social Media Professionalism (SMePROF) scale, developed and validated by Marelic et al.^13^ This scale consisted of 24 questions across seven facets of e-professionalism named: “ethical aspects, dangers of social media, excluding physicians, freedom of choice, importance of professionalism, physicians in the digital age, negative consequences.” The SMePROF scale was constructed and validated through a study involving 698 medical and dental students, using exploratory factor analysis and principal component analysis, with reliability indicated by a Cronbach’s alpha of 0.72.^13^ The original questionnaire was employed after obtaining written consent from the author. Participants responded to these questions using a five-point Likert Scale, where 1 indicated ‘Strongly Agree,’ 2 indicated ‘Agree,’ 3 indicated ‘Neutral,’ 4 indicated ‘Disagree,’ and 5 indicated ‘Strongly Disagree.’

### Statistical Analysis:

Data were analysed using SPSS version 26. Descriptive statistics were calculated for numerical variables (Mean ± SD) and categorical variables (Frequency, Percentage). Comparative analysis was done across two variables: gender (males vs females) and class seniority (Pre-clinical vs Clinical Classes). Comparisons of social media preference on a categorical scale were made using Chi-squared or Fisher’s exact test. Mann-Whitney U test was employed for Likert scale scores. A p-value of less than 0.05 was considered statistically significant.

## RESULTS

The survey received 252 responses out of 300 distributed forms (response rate 84%), of which 220 were included in the study following exclusions (valid response rate 73.3%) for withdrawn consent and improperly completed forms. Respondents, 46.4% male, and 53.6% female, had an average age of 21.82±1.59 years. They were spread across different academic years: second (24.1%), third (38.6%), fourth (19.1%), and final (18.2%).

The social media practices of medical undergraduates are detailed in Table-I. Most respondents preferred Instagram (82.3%), followed by Facebook (80%) and Snapchat (70.5%). Facebook usage was higher among males (87.3% vs 73.7%, p = 0.012), while Instagram was more prevalent among Pre-Clinical Year Students (87% vs 74.4%, p = 0.018). Time spent on social media averaged 5.5±4.38 hours. In comparison to females, males were more likely to display their own picture (94.1% vs 57.6%, p < 0.01) and post-work attire images (66.7% vs 43.2%, p< 0.001). Pre-Clinical Year Students were more likely to post workplace images (60.9% vs 42.7%, p = 0.002). Only a fraction of students (7.3%) posted disturbing content or accepted friend requests from patients.

**Table-I:** Social Media use practices.

Variable	Overall n (%)	Comparison by Gender			Comparison by Seniority		

		Males	Females	p-value	Pre-Clinical	Clinical	Sig.
***Social media platform***							
Facebook	176 (80%)	89 (87.3%)	87 (73.7%)	0.012*	105 (76.1%)	71 (86.6%)	0.060
Twitter	100 (45.5%)	43 (42.2%)	57 (48.3%)	0.361	69 (50%)	31 (37.8%)	0.079
Instagram	181 (82.3%)	83 (81.4%)	98 (83.1%)	0.745	120 (87%)	61 (74.4%)	0.018*
Snapchat	155 (70.5%)	69 (67.6%)	86 (72.9%)	0.396	96 (69.6%)	59 (72%)	0.708
Others	1 (0.5%)	1 (1%)	0 (0%)	0.464	1 (0.7%)	0 (0%)	0.440
Time spent on social media (hours)	5.5 ± 4.38	5.39 ± 5.23	5.59 ± 3.49	0.729	5.58 ± 5.24	5.35 ± 2.3	0.417
Own picture as display picture	164 (74.5%)	96 (94.1%)	68 (57.6%)	<0.001*	97 (70.3%)	67 (81.7%)	0.060
Post pictures work attire	119 (54.1%)	68 (66.7%)	51 (43.2%)	0.001*	69 (50%)	50 (61%)	0.114
Post pictures from workplace	119 (54.1%)	59 (57.8%)	60 (50.8%)	0.299	84 (60.9%)	35 (42.7%)	0.009*
Upload content that is disturbing for others	16 (7.3%)	5 (4.9%)	11 (9.3%)	0.208	12 (8.7%)	4 (4.9%)	0.292
Accept friend requests from patients	16 (7.3%)	5 (4.9%)	11 (9.3%)	0.009*	16 (11.6%)	6 (7.3%)	0.307

* Denotes significant difference (i.e., p < 0.05). Sig. / p-value of categorical variables calculated by Chi-squared test of independence and for numerical variables by Student t-test.

The responses to the SMePROF scale. Key findings are shown in Table-II.

**Table-II:** The distribution of student responses to the scale for measuring attitudes towards e-professionalism (SMePROF).

Q#	Theme	Question	Overall	COMPARISON BY GENDER			COMPARISON BY SENIORITY		

				Male	Female	p value	Pre-clinical	Clinical	p value
1	Ethical Aspects	Ethical for physicians to use social media in patient care.	3.24 ± 0.97	3.28 ± 0.97	3.19 ± 0.98	0.59	3.37 ± 0.98	3.01 ± 0.92	0.004*
2		Ethical to use personal accounts for patient interaction.	2.33 ± 1.01	2.36 ± 1.02	2.31 ± 1.01	0.605	2.43 ± 1.03	2.17 ± 0.97	0.038*
3		Social media can improve physician-patient communication.	3.47 ± 0.87	3.54 ± 0.82	3.42 ± 0.91	0.45	3.48 ± 0.89	3.46 ± 0.83	0.734
4		Social media doesn’t endanger physician-patient confidentiality.	3.2 ± 0.95	3.19 ± 0.91	3.2 ± 0.98	0.672	3.31 ± 0.91	3 ± 0.98	0.014*
5		Ethical for physicians to visit patient social media profiles.	2.3 ± 0.92	2.42 ± 0.85	2.2 ± 0.97	0.052	2.38 ± 0.93	2.17 ± 0.89	0.039*
6	Dangers of social media	Online info may affect hiring or interviews.	3.36 ± 1.01	3.36 ± 0.98	3.36 ± 1.04	0.828	3.46 ± 0.94	3.2 ± 1.11	0.113
7		Online behaviour may affect your professional reputation.	4 ± 0.73	3.94 ± 0.74	4.04 ± 0.72	0.293	4 ± 0.76	3.99 ± 0.68	0.699
8		Posts can lead to wrong assumptions about you.	3.88 ± 0.96	3.76 ± 0.97	3.97 ± 0.94	0.06	3.94 ± 0.96	3.77 ± 0.95	0.104
9		Online info may cost you your position or role.	3.37 ± 1.08	3.3 ± 1.08	3.42 ± 1.07	0.336	3.48 ± 1.01	3.18 ± 1.17	0.069
10		Inadmissible to share patient info without consent.	4.23 ± 0.92	4.21 ± 0.92	4.25 ± 0.92	0.62	4.25 ± 0.97	4.21 ± 0.81	0.257
11	Educating Physicians	Ban healthcare professionals from using social networks.	1.95 ± 0.85	1.84 ± 0.92	2.05 ± 0.78	0.018*	2.03 ± 0.89	1.83 ± 0.78	0.131
12		Restrict healthcare professionals on social networks.	2.18 ± 0.87	2.2 ± 0.96	2.16 ± 0.8	0.864	2.18 ± 0.9	2.17 ± 0.83	0.853
13	Freedom of Choice	I should have freedom to do anything online.	3.15 ± 1.12	3.11 ± 1.18	3.19 ± 1.07	0.547	3.28 ± 1.02	2.94 ± 1.25	0.034*
14		School/Admin can’t interfere with my online activities.	3.66 ± 0.95	3.69 ± 0.95	3.64 ± 0.96	0.753	3.65 ± 0.96	3.68 ± 0.95	0.656
15		Online activities don’t affect my professionalism.	3.55 ± 0.92	3.52 ± 0.96	3.58 ± 0.89	0.72	3.49 ± 0.91	3.65 ± 0.94	0.143
16	Importance of Professionalism	I comply with my professional behaviour expectations.	3.91 ± 0.71	3.95 ± 0.75	3.87 ± 0.67	0.354	3.93 ± 0.74	3.88 ± 0.66	0.435
17		I understand professional behaviour and expectations.	4 ± 0.64	3.96 ± 0.69	4.04 ± 0.59	0.537	3.96 ± 0.68	4.09 ± 0.55	0.214
18		Expect high-level professionalism from early student life.	3.35 ± 0.98	3.27 ± 0.96	3.41 ± 1.01	0.372	3.31 ± 1.05	3.4 ± 0.87	0.546
19	Physicians in Digital Age	Guiding patients to online info is responsibility of physician.	3.66 ± 0.93	3.75 ± 0.83	3.59 ± 1.02	0.468	3.69 ± 0.93	3.62 ± 0.94	0.593
20		As medical graduate, I should keep up with social media trends.	4.05 ± 0.82	4.19 ± 0.75	3.92 ± 0.85	0.022*	3.99 ± 0.82	4.15 ± 0.8	0.115
21		Teachers should counsel students on use of social media.	3.86 ± 0.89	3.94 ± 0.95	3.8 ± 0.82	0.084	3.78 ± 0.93	4.01 ± 0.79	0.072
22	Negative Consequences	Professionals can’t fully relax.	3.97 ± 1.04	3.98 ± 1.13	3.96 ± 0.96	0.48	3.84 ± 0.97	4.18 ± 1.14	0.001*
23		Social media have exposed professionals to public.	3.62 ± 2.81	3.53 ± 0.92	3.69 ± 3.74	0.211	3.75 ± 3.48	3.4 ± 0.87	0.725
24		Online professionalism is not always possible.	3.31 ± 0.98	3.25 ± 1.08	3.36 ± 0.88	0.632	3.35 ± 1	3.24 ± 0.94	0.445

* Denotes significant difference (i.e., p < 0.05). Sig. / p-value calculated by Mann-Whitney U test.

### Ethical Aspects:

Pre-Clinical students were more likely to agree with the use of social media for patient care (3.37 ± 0.98 vs 3.01 ± 0.92, p = 0.004) and interaction (2.43 ± 1.03 vs 2.17 ± 0.97, p = 0.038), and considered visiting patient profiles ethical (2.38 ± 0.93 vs 2.17 ± 0.89, p = 0.039). Pre-clinical students more strongly suggested that social media doesn’t endanger physician-patient confidentiality. (3.31 ± 0.91 vs 3 ± 0.98, p = 0.014).

### Educating Physicians:

There was a significant gender difference, with females more inclined to support a ban on healthcare professionals using social media (2.05 ± 0.78 vs 1.84 ± 0.92, p = 0.18).

### Freedom of Online Activities:

Pre-Clinical students had a stronger belief in the freedom to engage in online activities without interference (3.28 ± 1.02 vs 2.94 ± 1.25, p = 0.034).


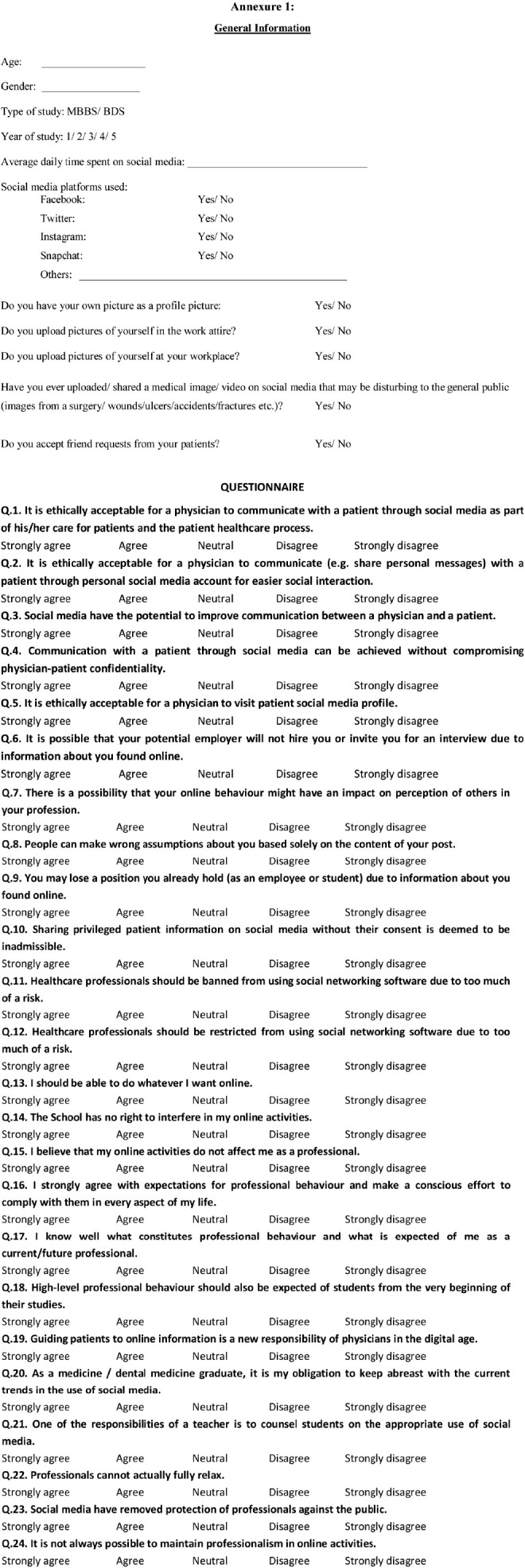


### Physicians in digital age:

Males were more in favour of keeping up with social media trends (4.19 ± 0.75 vs 3.92 ± 0.85, p = 0.022).

### Negative Consequences:

Clinical Year Students held a stronger opinion that professionals cannot fully relax online (4.18 ± 1.14 vs 3.84 ± 0.97, p = 0.001). For most other questions, responses varied insignificantly by gender or seniority.

## DISCUSSION

The findings of this study provide valuable insights into the social media practices and attitudes of undergraduate medical students toward e-professionalism. Our findings reveal that students are highly active on social media across various platforms, with Instagram being the most popular, followed by Facebook. These findings align with previous research conducted among medical and dental students in Croatia, which highlighted the popularity of Facebook followed by Instagram among this demographic.^14^

Further exploration in the current study showed that Facebook usage was higher among males vs females, while Instagram was more prevalent among pre-clinical year students vs clinical years. A review article in 2021, reported that social media usage trends vary widely by regional differences, level of seniority, and type of medical specialization.^3^ Thus, indicating the importance of exploring local social media usage trends along with associated factors to incorporate adequate educational interventions and policies.

Interestingly, the study found notable differences in social media practices based on gender and academic seniority. This study showed that male students were more likely to display their own pictures and post work attire images compared to their female counterparts. A previous study delineated that purpose of social media usage differs between genders: females mostly use social media for academic or professional purposes, whereas males mostly utilize social media for personal purposes.^8^ This gender disparity in posting behavior on social media platforms may be attributed to various factors, including self-presentation and professional identity formation. Additionally, pre-clinical year students were more inclined to share workplace images, possibly reflecting their eagerness to portray their early clinical experiences.

It is encouraging to note that only a fraction of students in this study reported posting disturbing content or accepting friend requests from patients. This suggests a responsible approach to maintaining professional boundaries on social media, as engaging in such behaviors can potentially compromise patient privacy and confidentiality. Similar studies conducted among dental students in Malaysia and Finland also reported a low prevalence of posting unprofessional content on social media platforms,^9^ indicating a general awareness of the importance of professionalism among healthcare students. On the other hand, a questionnaire-based survey among 512 Greek dental students reported that a majority of these students had posted unprofessional content on their social media profiles, as evidenced by 71.7% posting pictures from holidays, 41.5% from nightclubs, and 26.2% photographs wearing swimwear/underwear.^15^

The assessment of attitudes towards e-professionalism using the SMePROF scale revealed that Pre-clinical students demonstrated a greater inclination towards utilizing social media for patient care and interaction, as well as considering visiting patient profiles as ethical. This may be attributed to their exposure to theoretical knowledge and eagerness to explore digital platforms for professional purposes. On the other hand, clinical year students held a stronger belief that healthcare professionals cannot fully relax when online but need to maintain a cautious professional attire. This is possibly due to their exposure to real-world clinical scenarios and ethical dilemmas. Previously studies have reported a decreasing trend of social media usage for patient care and interaction with increasing age and seniority among healthcare professionals.^16^,^17^

Furthermore, the study identified gender-based differences in attitudes toward social media use. Female students were more likely to support a social media ban for healthcare professionals, possibly reflecting their concerns regarding the potential ethical and privacy risks associated with social media use. In contrast, male students exhibited greater interest in keeping up with social media trends, suggesting a desire to stay informed and connected in the digital sphere. The gender difference is also evident in previous studies exploring e-professionalism among medical students and healthcare professionals of various specialities.^3^,^10^,^18^ However, the nature of variation is subjected to regional and disciplinary contexts. For example, Irfan et al.^10^ and Wang et al.^19^ reported higher professional social media use among females, whereas Patel et al.^20^ and Gupta et al.^21^ found a predominance of males in professional contexts, particularly in radiology and orthopaedic surgery. While these findings suggest a notable gender difference, it’s important to consider that cultural contexts may influence these trends. These findings also highlight the need for tailored educational interventions and policies that address the specific concerns and perspectives of different genders.

It is important to note that the study did not find significant variations in attitudes towards e-professionalism based on academic seniority, gender, or other demographic factors for most of the questionnaire items. This suggests a general consensus among the participants regarding professionalism in the context of social media use. However, further research with a well distributed larger sample may provide deeper insights into these aspects. The results of this study may be generalized to similar settings and populations, particularly within medical schools in Pakistan. However, caution should be exercised when applying these findings to different cultural contexts or educational systems.

### Strengths:

However, despite these limitations, this study is unique in exploring the detailed social media usage trends among undergraduate medical students, while considering the demographic variables. Another, great strength of this study is employing the pre-validated SMePROF scale for the assessment of e-professionalism, as previously no such data is available in Pakistan.

The results underscore the importance of incorporating social media education and professionalism training into the medical curriculum to equip future healthcare professionals with the necessary skills and knowledge for responsible digital engagement. It is crucial to strike a balance between leveraging the benefits of social media platforms for professional growth and maintaining ethical standards to ensure patient privacy and professionalism in the digital age.

### Limitations:

This study is limited in exploring the attitudes of medical students towards e-professionalism because of its cross-sectional design. Also, the results reported in this study are self-reported by the students, which might have created response bias.

## CONCLUSION

This study highlights the widespread social media usage among undergraduate medical students and its impact on e-professionalism. Gender and academic seniority influence social media practices and attitudes towards e-professionalism. These findings emphasize the importance of targeted education and policies to promote e-professionalism in the healthcare field.

### Recommendations:

- Incorporating social media education into the medical curriculum is crucial for promoting responsible online behavior, upholding professionalism, and ensuring patient privacy in the digital age.
- Further research is needed to explore the long-term impact of social media on professional practice and develop comprehensive guidelines for ethical and responsible digital engagement in healthcare.
